# Exploring the presence of the *GPR126* single nucleotide polymorphism (rs536714306) in periodontal patients of European ancestry

**DOI:** 10.1186/s13104-025-07443-5

**Published:** 2025-08-22

**Authors:** Eirini Chatzopoulou, Galinos Fanourakis, Nikolaos Papanikolaou, Ioulia Chatzistamou, Xanthippi Dereka, Heleni Vastardis

**Affiliations:** 1 U.F.R. d’Odontologie, Université Paris Cité, 1 Rue Maurice Arnoux, Montrouge, Paris, 92120 France; 2 Service of Odontology, Periodontal and Oral Surgery Unit, Rothschild Hospital (AP-HP), 5 Rue Santerre, Paris, 75012 France; 3 Department of Oral Biology, School of Dentistry, National and Kapodistrian University of Athens, 2 Thivon Str, Goudi, Athens, 11527 Greece; 4 EnzyQuest PC, Science and Technology Park of Crete, 100 Nikolaou Plastira Str., Vassilika Vouton, Heraklion, 70013 Greece; 5 Department of Pathology, Microbiology and Immunology, School of Medicine, University of South Carolina, 6439 Garners Ferry Road Columbia, Columbia, SC 29209 USA; 6 Department of Periodontology, School of Dentistry, National and Kapodistrian University of Athens, 2 Thivon Str, Goudi, Athens, 11527 Greece; 7 Department of Orthodontics, School of Dentistry, National and Kapodistrian University of Athens, 2 Thivon Str, Goudi, Athens, 11527 Greece

**Keywords:** Periodontitis, *GPR126* gene, Polymorphism

## Abstract

**Objective:**

G protein-coupled receptor 126 (*GPR126*) gene has been implicated as a potential susceptibility factor for aggressive periodontitis in Japanese patients. This study aimed to investigate the presence of the *GPR126* [c.3086 G > A] (rs536714306) polymorphism in patients with periodontitis in a Greek population and periodontal cases of European ancestry. A total of 82 subjects were recruited: 53 patients periodontally compromised (P) and 29 healthy controls (H). GPR126 genotyping was performed using Sanger sequencing. Additionally, data from the Gene-Lifestyle Interactions in Dental Endpoints (GLIDE) consortium were included in this study.

**Results:**

No variants (rs536714306) in the *GPR126* gene were detected in any of the samples. The homozygous for the reference allele GG genotype was observed in 100% of participants across all groups examined. Absence of *GPR126* [c.3086 G > A] polymorphism indicates no association with susceptibility to periodontitis in a Greek cohort and periodontally compromised cases of European ancestry. This is the first focused report evaluating the presence of this polymorphism in periodontitis patients in a European population. Further genome-wide studies in larger sample and diverse populations are warranted to fully elucidate the potential role of *GPR126* polymorphisms in periodontal disease susceptibility.

## Background

Periodontitis is a complex inflammatory disease characterised by the progressive destruction of the supporting tissues around teeth and tooth loss if left untreated [1]. Apart from the well-established role of the periodontopathogens, individuals’ susceptibility to the periodontal disease may be genetically determined [2, 3]. A number of studies have been conducted to investigate the possible role of genetic polymorphisms in the pathogenesis of periodontitis, especially of genes of immunoregulatory molecules, such as interleukins, chemokines and membrane receptors [4].

G protein-coupled receptors (GPCRs) represent the largest and most diverse family of membrane receptors in vertebrates. GPCRs transduce a great variety of extracellular messages such as hormones, growth factors, neurotransmitters and sensory messages of light and odors and subsequently trigger second messenger cascade mechanisms from the adjacent microenvironment or by other cells [5]. These receptors are grouped in five distinct families, including glutamate, rhodopsin, adhesion, frizzled/taste2, and secretin [6]. The adhesion family of GPCRs (aGPCR) contains the typical heptahelical domain and is characterized by a conserved GPCR proteolytic site and a long extracellular glycosylated N-terminal region with adhesion-like motifs, which promote cell-to-cell and cell-to-matrix interactions [7].

GPR126, a member of the aGPCR family, is encoded by the *ADGRG6* gene, is located at 6q24.2, consists of 26 exons [8] and was first described by Fredriksson et al. [9]. The precise function of GPR126 is not yet fully understood, however it seems that this receptor participates in various developmental processes, including the formation of the segmental body plan, trabeculation of the developing heart and formation of the semicircular canals of the inner ear [10–12]. It is considered a determining factor in endothelial cell biology and angiogenesis, in stimulating vascular endothelial growth factor (VEGF) signaling [13]. Furthermore, GPR126 is required for myelination of nerve axons by Schwann cells [14]. Along with a significant number of G protein-coupled receptors family members [15], GPR126 was until recently considered an orphan receptor, but it has been shown that collagen type IV [16], laminin-211 [17] and the prion protein [18] are activating ligands of GPR126.

To date, several variations in the *GPR126* gene have been described by genome-wide association studies. Single nucleotide polymorphisms (SNPs) of *GPR126* have been implicated in adolescent idiopathic scoliosis [19], determination of body and trunk height [20], pulmonary function [21], Gorlin syndrome [22] and arthrogryposis multiplex congenital [23]. Moreover, a *GPR126* rs536714306 polymorphism was reported that may be associated with aggressive periodontitis in the Japanese population, indicating possible involvement with mechanisms regarding the homeostasis of the periodontal ligament tissues and suggesting that differences between ethnic populations may be present when it comes to periodontitis-related genetic factor [24].

Based on these findings, we designed a study to investigate whether the *GPR126* SNP (rs536714306) was present in Greek individuals with and without periodontitis. The results were then compared with previous genome-wide association studies (GWAS) focusing on cases of European ancestry.

## Methods

### Study design and sample collection

All subjects participating in this study were referred to the Postgraduate Clinic of the Department of Periodontology, School of Dentistry, National and Kapodistrian University of Athens. All ethical requirements have been fulfilled and all relevant documents are available. *Τhe Ethics and Research Committee* of the School of Dentistry approved the study protocol (196/01-11-2012). All participants signed an informed consent, according to the general recommendations of the Declaration of Helsinki.

Participants were recruited from 2013 to 2015. They were classified according to the 2018 classification of periodontitis [25] and assigned into two groups: periodontally healthy individuals (H) and individuals with periodontitis (stages I, II, III and IV) (P). All participants were of Caucasian/Greek origin, non smokers and systemically healthy.

Gingival tissue samples and oral mucosa swab samples were obtained from the subjects and were processed for genomic DNA analysis. Briefly, 21 P patients and 12 H subjects, provided gingival tissue samples during conventional periodontal surgery and crown lengthening surgical procedure, respectively. We further recruited 32 P patients and 17 H subjects, who all provided an oral mucosa swab sample, by using a cotton stick against the buccal mucosa for 1 min. Gingival tissue samples were collected during routine periodontal surgical procedures to avoid additional invasive sampling, while buccal mucosa swabs were used in patients not undergoing surgery, in order to provide a non-invasive method for genetic analysis. It is noteworthy that both biological materials (i.e. gingival tissue and oral mucosa swabs) yielded high-quality genomic DNA, suitable for downstream molecular techniques and that the DNA source did not impact the detection of the SNP under investigation [26, 27].

### Genotyping

DNA was extracted with the DNeasy Blood and Tissue kit (Qiagen, Hilden, Germany) following manufacturer’s instructions. The DNA concentration was estimated by measurement of OD260 using a spectrophotometer (BioSpec-nano, Shimadzu, Japan). The target sequence of 454 bp was amplified, using previously reported primers [24]. Polymerase chain reaction (PCR) was carried out in a total volume of 25µL, containing 100ng genomic DNA, 5 pmol of each primer, 0.2 mM of each dNTP, 2.0mM of MgCl2, 2 µL of 10x PCR buffer and 0.5 U Taq DNA polymerase (KAPA Biosystems). Cycling conditions were 2 min at 95 °C followed by 40 cycles of 30 s at 95 °C, 30 s at 57 °C and 40 s at 72 °C, with a final extension of 72 °C for 7 min. PCR products were purified with a PureLink PCR purification kit (Invitrogen Life Technologies).

### Direct sequencing

Direct sequencing was carried out on purified PCR products. Sequence data were visualized with Chromas software (version 2.6.4) and analyzed using the Basic Local Alignment Search Tool (BLAST) from the National Center for Biotechnology (NCBI).

### Data sources

The Gene-Lifestyle Interactions in Dental Endpoints (GLIDE) consortium [28] is a unique collection of epidemiological cohorts with detailed information on clinical diseases and summary statistics for periodontitis. The study, among others, includes 17,353 cases and 28,210 controls, all from European ancestry.

## Results

**Fig. 1:**
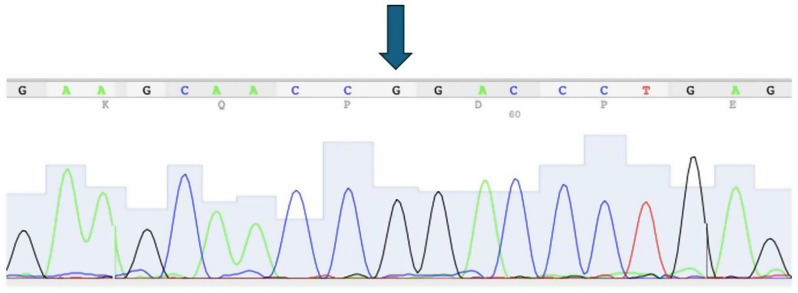
Sequence chromatogram

In the present study, 53 patients were diagnosed with periodontitis, and 29 individuals were periodontally healthy. Gender proportion was similar in all study groups. In the total of the 82 participants, the minor allele A was absent in all samples. The homozygous GG genotype (Fig. 1) was observed in 100% of P and H groups (Table 1).

Similar results were obtained when exploring the GLIDE database, indicating that a correlation between the development of periodontitis and the presence of the GPR126 rs536714306 polymorphism could not be established, at least in European populations.

The arrow indicates the G allele of the GG genotype of *GPR126* [c.3086 G > A] in a P patient.

**Table 1:** Frequency distribution of alleles

	*GPR126* [c.3086] GG genotype	*GPR126* [c.3086] GA / AA genotype
Periodontitis (*n* = 53)	53 (100%)	0
Healthy periodontium (*n* = 29)	29 (100%)	0

## Discussion

The precise role of *GPR126* in periodontal tissues remains unclear. In vitro studies have shown that wild-type GPR126 significantly increases the mRNA expression of bone sialoprotein, osteopontin, and Runx2 genes [24]. This effect is mediated through GPR126-induced upregulation of bone morphogenetic protein-2, inhibitor of DNA binding (ID) 2, and ID4 expression. These findings suggest that GPR126 may play a crucial role in maintaining periodontal ligament tissue homeostasis through the cytodifferentiation of human periodontal ligament (HPDL) cells [24]. Notably, the rs536714306 variant appears to negate these effects. In addition, GPR126’s unique combination of adhesion domains, including CUB (C1r-C1s, Uegf and Bmp1) and pentraxin domains [29], which are components of complement activation and acute phase plasma proteins respectively, suggests a potential role in innate immunity and inflammation [30]. This multifaceted functionality underscores the complexity of GPR126’s potential involvement in periodontal health and disease.

The present study investigated the potential association between the *GPR126* SNP rs536714306 [c.3086 G > A] and periodontitis in a Greek cohort. This missense SNP, located in exon 20, results in an arginine to glutamine substitution at position 1029 between transmembrane helices V and VI. Our analysis revealed no SNP alterations in any of the samples, with the homozygous for the reference allele GG genotype consistently present across all groups (P and H). These findings contrast with those of Kitagaki et al. [24], who reported a minor A allele frequency of 2.44% in Japanese patients with aggressive periodontitis, compared to 0.27% in controls.

Our research work represents the first examination focusing specifically on the *GPR126* SNP in a European population with periodontitis. An analysis of the NCBI dbSNP repository (https://www.ncbi.nlm.nih.gov/snp/rs536714306) revealed intriguing population differences in the prevalence of this variant. East Asian populations, particularly Japanese and Korean, show a higher prevalence of the variant compared to European and mixed populations. The variant appears to be extremely rare or absent in populations of African descent. The discrepancies between our findings and those in Asian populations highlight the importance of considering ethnic backgrounds in genetic risk factors for periodontitis. As some genes identified in Western genome-wide association studies are not detected in Asian GWAS, there may be ethnicity-specific genetic risk factors for the initiation and progression of periodontitis [24, 31, 32].

This study has potential limitations. A larger pool of periodontal compromised patients and healthy subjects would support our finding, if verified. Moreover, our analysis of the *GPR126* gene could have been extended throughout the whole gene and not be limited in detecting the rs536714306 polymorphism, with possible outcome the discovery of one or more polymorphisms, since *GPR126* seems to be involved in mechanisms that could affect the periodontal health.

However, while our sample size is relatively small, this study provides solid, albeit negative, results. These findings contribute to the emerging field of precision dentistry, which aims to tailor diagnostics and therapy to individual patients. By leveraging diverse data sources and rigorously testing prediction models, we can move towards more personalized oral healthcare [33].

## Conclusions

In conclusion, although this study found no presence of the *GPR126* SNP rs536714306 in periodontal patients in our Greek cohort, nor in periodontal cases of European ancestry, it serves as an additional step towards further investigation of *GPR126* rs536714306 in diverse populations worldwide. Extensive genome-wide association studies in Caucasian populations are warranted to further explore the potential role of this polymorphism in periodontal pathogenesis and its association with periodontal tissues.

## Data Availability

Sequence data that support the findings of the current study are available in the GenBank repository, GenBank accession numbers PQ509770-PQ509851.

## Abbreviations

GPCRs: G protein-coupled receptors
GPR126: G protein-coupled receptor 126
VEGF: Vascular endothelial growth factor
SNP: Single nucleotide polymorphism
GLIDE: Gene-Lifestyle Interactions in Dental Endpoints
H: Periodontally healthy individuals
P: Periodontal patients
PCR: Polymerase chain reaction
BLAST: Basic Local Alignment Search Tool
NCBI: National Center for Biotechnology
GWAS: Genome-wide association studies
